# Hints for Young Doctors

**Published:** 1854-10

**Authors:** C. D. Griswold

**Affiliations:** New York


Hints for Young Doctors. By C. D. Griswold, M. D., of New York.—For ten years I have led a somewhat variable and busy life, always devoted to the interests of my patients—when I had them to care for—and my profession: yet notwithstanding my predelictions for the use of the pen, I have seldom contributed anything of my experience, or inexperience, to strictly professional journals; preferring always to read for my own instruction, rather than to write for the information of others. The principle is wrong, although it is better to be silent than to affect to be overwise.

How much more attentively we watch the different phases and behaviour of a disease, when it is our intention to report it—how much more definitely each symptom is impressed upon the memory; and with what readiness its stages and the treatment may be recalled at any time afterwards. In this way a habit becomes confirmed, and holds good in all cases. In traveling formerly, I noticed everything for the purpose of giving a description. Nauvoo Temple has long since been crumbled to the earth; yet the peculiarities of that structure, and the ground and beautiful scene

I looked out upon from its tower, are still distinctly visible in my recollection. The habit of observation thus formed, has led me ever since to the upper deck of a steamboat, or the top of a stage coach, that I might look out; and to detest cars, because they shut me up.

There is more *utility* in this habit of close observation, than most physicians are inclined to acknowledge in practice. I can remember distinctly the details of cases that I attended years ago, with the modifications in the treatment to meet indications—I held in view, and do still, intend to publish them—and yet I have by no means a retentive memory upon general subjects.

This habit any one can acquire by proper discipline, and it is one exceedingly important to the physician. The young physician who writes out his cases, will be most sure to read the reports of others, and in this way his experience will be trebled in value, besides most likely escape that worst of all obstacles to progress, routine habits of practice.

In reading your Journal, which I always do with interest, I seldom pass over the report of a case; and if I know it to be from the pen of a young physician, I peruse it with a sort of "do by others as you would that they should do by you" principle—respect and encourage them by reading their productions, as we would cherish the memory of one departed—although both alike are obvious to our good intentions. In this way we not unfrequently fall in with good ideas, which like seed sown, spring up at a future time and multiply.

As I shall have no room in this for a "report," as I had intended, I will add one other hint for such of your readers as may be younger than myself, and put off the "case" to another day—or rather night.

In the first place, write out all your important cases; if time will not admit its being done immediately, keep thinking them over with that intention. When this is done, if you find any of them to contain facts which you believe to be of value, send them to a publisher, post paid. Do not make the mistake that many young writers do, by sending to the largest and most important Journals, for such are usually supplied with more matter than they can print; and therefore, in all probability, in such a case your production would never find a place in their pages, and you would most likely get discouraged with the first attempt. On the contrary, send your articles to a small Journal first, or to a new one that has little patronage—of which there an abundance thankful for small favors; and in order that you may be sure to see them if printed, it is a good plan to enclose the subscription price with the production, and but very few if any comments, aside from your name and address plainly written. Draw no inferences on your cases—your readers will do that, and save you your time, and paper, and likely enough no small amount of future regrets—but simply the medical facts, plainly and concisely stated. Remember, if you have any desire to see your article reprinted in other Journals, that it never will be if *long*. Follow these rules perseveringly, and you will ultimately not only succeed as authors, but as good physicians.

A *regular* medical man told me, not long ago, that he subscribed for but one Journal—and that I will not mention—which he never found time to read. Now I shall remember this man as long as I do the Nauvoo Temple, for I have a habit of remembering such "cases." I shall never apprehend, on meeting him, that he has seen this comment, for did he read this Journal he would know better than to make such a statement.

I remember calling on Dr. John W. Francis, late one evening, and finding him in bed, he not being very well; yet his light was safely arranged, and within reach there was reading matter enough to last all night. If there is no other time, an hour may be spent every night in reading, before the eyelids drop; and he who cultivates his intelligence, as a physician should, will improve even this hour, if he has no other. If you do not read, never tell of it, for it is more creditable by far to have time for this, than too much business.—*Boston Med. and Surg. Journal.*

